# Perspectives on Early Screening and Prompt Intervention to Identify and Treat Maternal Perinatal Mental Health. Protocol for a Prospective Multicenter Study in Italy

**DOI:** 10.3389/fpsyg.2020.00365

**Published:** 2020-03-11

**Authors:** Loredana Cena, Gabriella Palumbo, Fiorino Mirabella, Antonella Gigantesco, Alberto Stefana, Alice Trainini, Nella Tralli, Antonio Imbasciati

**Affiliations:** ^1^Department of Clinical and Experimental Sciences, Section of Clinical Psychology, Observatory of Perinatal Clinical Psychology, University of Brescia, Brescia, Italy; ^2^Center for Behavioural Sciences and Mental Health, National Institutes of Health, Rome, Italy

**Keywords:** perinatal, antenatal, postnatal, anxiety, depression, screening, assessment, psychological treatment

## Abstract

**Background:**

The most common mental disorders in women during the perinatal (antenatal and postnatal) period are depressive syndromes and anxiety syndromes. The global prevalence of maternal perinatal depression ranges from 10 to 20%, while the prevalence of perinatal anxiety ranges from 10 to 24%. The comorbidity of mood and anxiety disorders in perinatal women is common, reaching 40%. In Italy, a few studies have been undertaken to evaluate the prevalence of perinatal depression and anxiety, and there is still a scarcity of research and intervention programs regarding primary prevention. Three of the main aims of this study are: (1) to evaluate the prevalence of maternal perinatal depression and anxiety in a large sample of women attending healthcare centers in Italy; (2) to investigate the psychosocial risks and protective factors associated with maternal perinatal depression and anxiety; (3) to evaluate the effectiveness of a manualized psychological intervention (Milgrom et al., 1999) to treat perinatal depression; (4) to evaluate the psychometric properties of both the Edinburgh Postnatal Depression Scale and the Patient Health Questionnaire-9 in detecting perinatal depression; and (5) to evaluate the influence of maternal depression and anxiety on the development of infant temperament.

**Methods:**

This is a prospective cohort study, which merges an observational design and a pre-post intervention design. The study includes a 1-year recruitment period and a one-year follow-up period. The methodological strategy includes: (1) self-report questionnaires on maternal depression, anxiety, health status, quality of life and psychosocial risks; (2) a self-report questionnaire to measure the infant’s temperament; (3) a clinical interview; (4) a structured diagnostic interview; and (5) a psychological intervention.

**Discussion:**

The results of this study may contribute to our knowledge about prevalence of antenatal and postnatal depression and anxiety (during both the trimesters of pregnancy and the first six trimesters after birth) and about the effectiveness of early psychological intervention in the perinatal health services.

## Introduction

### Background

The perinatal period is a complex and vulnerable period covering the prenatal and postnatal period, which presents a series of challenges for women and men who are on their journey toward parenthood (Parfitt and Ayers, 2014). This transition can be a happy phase of life, full of positive expectations for the parents-to-be; however, it can also be a source of distress and difficulties. Indeed, it is common for pregnant women and new mothers to experience variations in mood and emotional changes due to a combination of hormonal factors and the burden of maternal responsibilities. Typically, this situation tends to resolve itself naturally, however, where there is a wide gap between expectations or resources and the reality of motherhood, psychological difficulties or mental health disorders can be consolidated or even worsen (Sipsma et al., 2016). Moreover, this situation has consequences on a larger scale because, during the perinatal period, the wellbeing and mental health of the mother, father and baby are closely interrelated (Yeaton-Massey and Herrero, 2019). Indeed, parental perinatal complications can interfere with the parent-child relationship, with the risk of significant consequences over the years (Cirulli et al., 2003; Milgrom et al., 2006; Meneghetti, 2007, 2011; Branchi et al., 2013; Stefana and Lavelli, 2017) for the child’s cognitive, social and emotional development (Kim-Cohen et al., 2005). With regards to the child, it must be taken into account that perinatal clinical psychology studies (Imbasciati et al., 2007; Imbasciati and Cena, 2015a, b) and neuroscience research (Imbasciati and Cena, 2017) show that the mother–baby–father relationships influence the construction of the child’s neuropsychic structure in his brain, especially during the perinatal period (considered in our study to be the first 1000 days of life: from conception to 18 months of age) and, to an extent, throughout life (Imbasciati, 2006; Imbasciati and Cena, 2018).

The most common perinatal mental disorders in women during pregnancy and postpartum are depressive syndromes and anxiety syndromes: non-psychotic disorders characterized by the specific feelings and thoughts about the parental role (Robinson and Stewart, 2001). The global prevalence of clinically significant maternal perinatal depression ranges from 10 to 20% (Korja et al., 2018; Earls et al., 2019), while the prevalence of perinatal anxiety disorders ranges from 10 to 24% (Dennis et al., 2018; Yeaton-Massey and Herrero, 2019). Furthermore, the comorbidity of mood disorder and anxiety disorder in perinatal women is common (Falah-Hassani et al., 2016), reaching 40% in some studies (Reck et al., 2008; Austin et al., 2010), but often under-diagnosed (Misri and Swift, 2015). In Italy, the few studies carried out to evaluate the prevalence of perinatal depression and anxiety show large variability. Evaluations of maternal perinatal depression and anxiety record ranges of 1.6–26.6% and 6.4–20.5%, respectively (Currò et al., 2009; Mauri et al., 2010; Banti et al., 2011; Girardi et al., 2011; Giardinelli et al., 2012; Elisei et al., 2013; Palumbo et al., 2016; Clavenna et al., 2017; Di Venanzio et al., 2017; Vizzini et al., 2018). The above-mentioned data are influenced by factors such as different screening tools (which have different psychometric properties) and time periods.

Most studies in the literature undertaken by the Italian National Institute of Health and funded by the Ministry of Health – National Centre for Disease Prevention and Control (Palumbo et al., 2016, 2017, 2018) have focused mainly on the effects of maternal postnatal mental health complications. However, more recent studies (Kendig et al., 2017) have examined maternal antenatal and postnatal depression and anxiety, to understand better their short- and long-term effects on both mother and fetus/child (Dunkel Schetter and Tanner, 2012; Suri et al., 2014; Glover, 2015; van der Waerden et al., 2015; Riva Crugnola et al., 2016; Galbally and Lewis, 2017; Field, 2018; Shonkoff et al., 2019).

However, although several recent studies suggest the importance of screening early on in pregnancy in order to reduce anxiety or depressive symptoms and prevent a postpartum episode (Austin, 2004; O’Connor et al., 2016), there are still few research and intervention programs regarding primary prevention. These studies suggest the importance of identifying perinatal mental complications from the beginning of the pregnancy in order to reduce symptoms and prevent a postpartum episode through developing pathways tailored to safeguard the health of mother and child. Nevertheless, in Italy, such programs are not adequately integrated with clinical guidelines for appropriate practical care planning routines. This is due partially to the absence of an Italian policy to screen for anxiety and/or depression during the perinatal period. Additionally, there is limited training among healthcare providers on how to choose the most appropriate screening tool and the cut-off for the specific period.

Through this study, the Observatory of Perinatal Clinical Psychology (University of Brescia, Italy) and the Italian National Institute of Health aim to help promoting and improving the perinatal mental health prevention and early intervention programs in the Italian health service.

### Main Objectives

(1) In light of the above, the first objective of this study is to evaluate the prevalence of both maternal antepartum and postpartum depression and anxiety in a large sample of women attending healthcare centers in Italy.
(2) The second objective, parallel to the first, is to investigate the psychosocial risks and protective factors associated with maternal antepartum and postpartum depression and anxiety.
(3) The third objective, building on the first, is to evaluate the psychometric properties of both the Edinburgh Postnatal Depression Scale (Cox et al., 1987) and the Patient Health Questionnaire-9 (Kroenke et al., 2001) in detecting perinatal depression.
(4) The fourth objective is to evaluate the effectiveness of a manualized psychological intervention (Milgrom et al., 1999) for both antenatal and postnatal depression in a large sample of mothers in Italy.
(5) The final objective is to evaluate the influence of maternal depression and anxiety on the development of baby temperament during a period of a minimum of 12 months up to a maximum of 18 months from birth.

### Hypotheses

(1) On the basis of the current literature (Underwood et al., 2016; Okagbue et al., 2017; Hahn-Holbrook et al., 2018), we expect to find a prevalence of perinatal depression within the range of 13–18%, with higher rates during pregnancy than in the first year following childbirth.
(2) Based on previous research (Ghaedrahmati et al., 2017; Leach et al., 2017), we assume that the factors associated with a higher risk of perinatal depression and anxiety will include previous history of depression and anxiety, a negative attitude toward a recent previous pregnancy, high-risk pregnancy, young maternal age, limited social support, and financial problems.
(3) In accordance with previous studies on screening for antenatal or postnatal depression (Yawn et al., 2009; Zhong et al., 2014; Santos et al., 2016), we expect that both the Edinburgh Postnatal Depression Scale and the Patient Health Questionnaire-9 will be reliable and valid scales for the assessment of perinatal depression.
(4) Based on a prior Italian study (Mirabella et al., 2016) showing the effectiveness of Jeannette Milgrom’s (Milgrom et al., 1999) psychological intervention among women suffering from postpartum depression, we assume that intervention can be effective also with antepartum depressed women.
(5) Given that the literature (Erickson et al., 2017) has offered ambiguous results on the association between prenatal maternal mental health and the development of infant temperament, we have no specific assumptions to this regard.

## Methods and Analysis

### Study Design

This is a prospective cohort study which merges an observational design and a pre–post intervention design. The study involves a one-year recruitment period and a one-year follow up period. The methodological strategy includes: self-report questionnaires on maternal depression, anxiety, health status, quality of life, and psychosocial risks; a self-report questionnaire to measure the infant’s temperament; a clinical interview; a structured diagnostic interview; and a psychological intervention.

### The Observatory of Perinatal Clinical Psychology

The Observatory of Perinatal Clinical Psychology, established at the Faculty of Medicine and Surgery of the University of Brescia (Italy) and affiliated to the Department of Clinical and Experimental Sciences, is an authorized center for perinatal studies and research, with contributions by professionals and researchers who deal with perinatal clinical psychology. The interdisciplinary collaboration network and the clinical and experimental research projects undertaken have the primary objective of protecting and promoting the mental health of women, men, couples, children and families. All this has also formed the subject of postgraduate course in Perinatal Clinical Psychology organized annually at the University of Brescia since 2012 (Scientific Directors: Imbasciati, Cena, 2012–2019)^1^ and attended by Italian professionals employed in Institutions responsible for providing perinatal support (e.g. clinics, hospitals, local health authorities).

### Reference Centre for Behavioural Sciences and Mental Health – National Institute of Health

The Italian National Institute of Health (Istituto Superiore di Sanità, ISS) is the main Italian research institute in the field of biomedicine and public health. It is the technical and scientific body of the Italian National Health Service. Mission: Promotion and protection of national and international public health through research, surveillance, regulation, control, prevention, communication, counseling and training. Vision: The ISS produces knowledge through research and trials and disseminates scientific knowledge and evidence to decision-makers, professional workers and citizens in order to protect and promote public health.

There is no health without mental health. This is the basis for the activity of the Reference Centre for Behavioural Sciences and Mental Health (SCIC), whose mission, in line with the 2013–2020 Mental Health Action Plan of the WHO, is to promote research and its application to improve the health of people suffering from mental disorders, their families and the general population.

### Selection of Expert Healthcare Centers

All participants of the past editions of the aforementioned course are contacted to gauge their center’s interest in taking part in the study. Once the voluntary and free applications are collected, we proceed with the selection of those healthcare centers which meet the necessary criteria (including the presence of at least one experienced psychotherapist). The subsequent step is to send a letter to the directors of the identified centers in order to request their participation in the project. The result is that 11 centers throughout Italy take part in the study (see Table 1). In each participating healthcare center a coordinator (an experienced psychologist–psychotherapist or a child neuropsychiatrist) is identified to be responsible for set up a scientific technical committee to prepare and plan the research project adapted to their own operational reality. Coordinators are responsible for identification, recruitment, data collection, intervention and follow-up of study patients.

**TABLE 1:** Healthcare Centers Involved in the Study.

**Location**	**Name**	**Unit type**	**Promotion of perinatal mental health awareness**	**Professionals involved**
Treviolo (Bergamo)	Mani di Scorta	Clinic and Family Centre	Individual or group antenatal meetings	1 PsyD 1 Midwife
Bologna	LHA of Bologna Child and Adolescent Neuropsychiatry (Mental Health Department – Pathological Addictions)	Neuropsychiatry of Infancy and Adolescence (Mental Health Department – Pathological Addictions) in the NICU (Mother and Baby Department)	Individual meetings during the infant’s stay in the NICU	1CN 1 PsyD 1 PsyD in training
Bologna	Maggiore Hospital	Normal Pregnancy and Breastfeeding Department	Individual or group antenatal meetings	1 PsyD 1 Gynecologist 2 Midwives
Brescia	Clinical Institute City of Brescia	Obstetrics and Gynecology OU	Group antenatal meetings and individual meetings postpartum	2 PsyD 1 PT in training
Enna	Umberto I Hospital	Obstetrics and Gynecology OUC, Normal Pregnancy Clinic	Individual antenatal meetings	1 PsyD
Florence	LHA of Toscana Centro	Family Clinic and Pediatric Surgery	Group antenatal meetings	4 PsyD
Mantua	Carlo Poma Hospital	Clinical Psychology Department; NICU	Group antenatal meetings and individual meetings during the infant’s stay in the NICU	1 PsyD 1 Midwife 1 Nurse
Milan	San Giuseppe Hospital	Obstetrics and Gynecology OU	Group antenatal meetings	1 PsyD
Novara	GruppoPsychè Association, Maggiore della Carità Hospital	Obstetrics and Gynecology OU	Group antenatal meetings	3 PsyD
Rome	Cristo Re Hospital	Obstetrics and Gynecology OUC	Group antenatal meetings	5 PsyD 1 Psychologist
Collegno (Turin)	LHA 3 of Turin	Assistive Process, Perinatal Psychology, Specialist Clinic of Perinatal Psychology, and Vaccine Clinic	Individual or group meetings postpartum	1 PsyD 1 Psychologist

*CN = Child Neuropsychiatrist; LHA = Local Health Authority; NICU = Neonatal Intensive Care Unit; OU = Unit/Department; OUC = Operating Unit Complex; PsyD = Psychologist–Psychotherapist; RHS = Regional Health Service.*

### Patient and Public Involvement

Patients and public are not involved in the design or recruitment of other participants of this study. However patients are considered in adapting the intervention.

### Setting

All mothers or pregnant women who attend one of the selected healthcare centers are invited to participate in the study. The data collection was initiated in June 2017 and will continue until November 2019.

### Training Program for Healthcare Professionals

Several meetings with the participating healthcare centers take place to plan and share the logistics and the project organization with the professionals involved.

The Observatory of Perinatal Clinical Psychology, thanks to the collaboration with the Italian National Institute of Health, forms all of the healthcare professionals who are involved in the study through two specialist training courses.

The first course is 1 day of face-to-face training focused on the promotion of perinatal mental health awareness and screening. The following topics are covered: methodologies, tools, theoretical and applicative study material, to be used during the clinical practice to identify the risks of perinatal anxiety and depression. The educational objectives are: to improve knowledge of the main theories on perinatal depression and anxiety; to improve knowledge about perinatal screening procedures, to improve maternal awareness regarding perinatal depression and anxiety and to screen and evaluate the psychosocial risk factors. A further 3-day face-to-face training course is provided to the psychotherapists only, and focused on Jeannette Milgrom’s psychological intervention for perinatal depression and anxiety (Milgrom et al., 1999, 2015a,b; Milgrom and Gemmil, 2015). Particular attention is also focused on how to conduct a clinical interview in perinatal populations. The course topics are: understanding the main elements and characteristics of the intervention program and the management of perinatal depression and anxiety; perinatal depression and anxiety assessment; clinical interviews with mothers; and application of the psychological intervention to mothers, also involving the babies and fathers.

During the study, there is regular contact between the coordinating center (the Observatory of Perinatal Clinical Psychology), the Italian National Institute of Health and the eleven participating healthcare units in Italy. Monthly Skype conference calls, Webex teleconferences, telephone consultations and periodic meetings are organized to keep all participants abreast of project progress and the stages of recruitment.

### Selection Criteria

#### Inclusion Criteria

Mothers are eligible for inclusion in the study if they meet the following criteria: (1a) they are pregnant or (1b) they have a biological newborn aged ≤6 months; (2) they are able to speak and read Italian.

#### Exclusion Criteria

Mothers are excluded if (1) they have psychotic symptoms; (2) they exhibit non-suicidal self-harming or suicidal behavior; (3) they have issues with drug or substance abuse.

### Procedures

**FIGURE 1:**
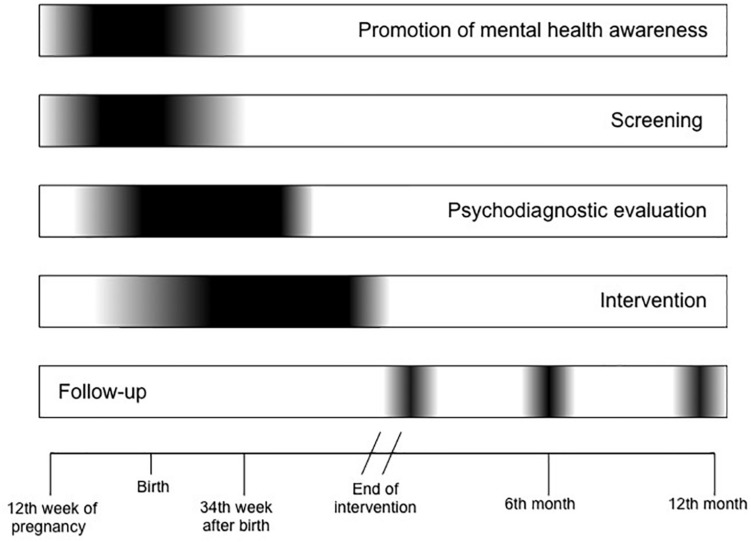
Timeline.

This five-phase study comprises a perinatal mental health awareness period, a screening period (up to the baby’s first vaccination), a psychodiagnostic evaluation, an intervention period (10 weeks’ duration), and a post-intervention follow-up (up to 12 months from the end of intervention). The timeline of the whole procedure is depicted in Figure 1.

#### Phase 1: Promoting Perinatal Mental Health Awareness

All mothers assisted in the healthcare centers are informed about the perinatal mental health problems that can occur during pregnancy and postpartum, and their impact on both the mother’s health and the baby’s development. Subsequently, mothers are invited to participate in the perinatal depression screening.

#### Phase 2: Screening for Psychosocial Risks, Depression, and Anxiety

Screening is performed once during the pre- or post-partum period, depending on the characteristics of each healthcare center. All mothers complete the Psychosocial and Clinical Assessment Form (Mirabella et al., 2016), the Italian versions of both the Edinburgh Postnatal Depression Scale (EPDS) (Cox et al., 1987; Benvenuti et al., 1999) and the Patient Health Questionnaire-9 (PHQ-9) (Kroenke et al., 2001) to evaluate depressive symptoms and the Italian version of the State-Trait Anxiety Inventory (STAI) (Spielberger et al., 1983; Pedrabissi and Santinello, 1992) to evaluate anxiety.

Mothers are required to complete the psychodiagnostic evaluation within one week of the date of positive screening results.

#### Phase 3: Psychodiagnostic Evaluation

All mothers undergo an interview with a clinical psychologist. Within the following week, in the case of clinician-generated diagnosis of perinatal depression and/or anxiety (according to the DSM-5 criteria), these mothers are further assessed using the Mini-International Neuropsychiatric Interview (MINI) Plus (Lecrubier et al., 1997; Sheehan et al., 1998) to confirm the diagnosis, the World Health Organization Quality of Life (WHOQOL) BREF (The Whoqol Group, 1998) to assess the quality of life and the Italian Questionnaires of Temperament (QUITs) (Axia, 2002) to assess the babies’ traits in temperament.

All mothers receive verbal feedback about the results of the psychodiagnostic evaluation. Depressed and/or anxious mothers are invited to participate in the intervention phase of the study. Whereas mothers with postpartum psychotic disorders, non-suicidal self-harm tendencies or suicidal ideation are sent to a psychiatric service and excluded from the study.

#### Phase 4: Intervention

The intervention is based on the model developed by Milgrom at the Australian Parent-Infant Research Institute (PIRI) at Heidelberg Repatriation Hospital (Milgrom et al., 1999, 2011, 2015a,b; Milgrom and Gemmil, 2015) to reduce maternal prenatal and postnatal depression, anxiety and parenting difficulties. Moreover, this model provides for the simultaneous assessment of the neurobiological, psychological and social factors that contribute to developing psychological vulnerability in the perinatal period.

Milgrom developed a psychosocial and psychological treatment by adapting Cognitive-Behavioral Therapy (CBT) interventions for women suffering from depression both in pregnancy and postpartum (Milgrom et al., 1999; Milgrom and Gemmil, 2015). The effectiveness of this treatment has been assessed in both short- and long-term application, and its validity has been demonstrated and compared to pharmacological therapy using antidepressants. It has been deemed more effective than drugs in maintaining long-term results (Milgrom et al., 2015a).

The intervention consists of group-based CBT and focuses on the women’s life events and mood and on practical issues. The intervention takes place primarily in groups because the group setting allows participants to meet, compare and share their experiences, offering mutual support. However, in the event of impossibility of organizing a group of at least three participants within a few weeks of the psychodiagnostic evaluation, or where a participant is unable to take part regularly in weekly group sessions due to health or organizational reasons, each healthcare center offer the option of individual treatment. This solution allows a more flexible approach than group treatment, since the program can be adapted to the specific situation of the woman in question. In both cases, information and activity material are distributed between sessions.

This clinic-based group intervention is led and facilitated by a licensed psychotherapist and consists of ten weekly sessions of 90 min exclusively dedicated to the pregnant women/new mothers, three sessions involving the partners/fathers, and another three sessions dedicated to mother–child interaction. [In our study, before this last module, the QUITs (Axia and Weisner, 2002) is administered to assess infant temperament traits]. The content of the individual counseling is the same as that of group-based counseling and consists of ten sessions of 60 min. At the end of each session, participants receive information and activity materials to be used in the next session. Between sessions, participants are encouraged to put what they have learnt/experienced during the treatment into practice in everyday life. The session program is shown in Table 2.

**TABLE 2:** Treatment Program.

**Step 1: Behavioral interventions**	

Session 1	**Understanding and dealing with depression.** *The sessions involve the individual or group development of the themes of the treatment program. The first session begins with participant and the therapist introducing themselves, sharing experiences to encourage a sense of belonging within the group. This psychoeducational session invites women describe their expectations of motherhood and the therapist provides information on prenatal and postnatal depression*.
Session 2	**Pleasurable activities: where do I find the time?** *The therapist encourages the women to think about their daily routine and to talk about their difficulties, thoughts, emotions and joys. Possible pleasurable activities (to enjoy alone, with their child and with their partner) are suggested for reducing the stress and fatigue of everyday commitments*.
Session 3	**Quick relaxation strategies.** *The therapist proposes some relaxation techniques (for example, Jacobson’s progressive muscle relaxation). Together with participants, the therapist examines possible simple “stress-eliminating” techniques*.
Session 4	**Assertiveness and self-esteem: telling others what I think and how I feel.** *The therapist proposes role-playing and communication techniques intended to encourage the women to express their needs in an assertive manner*.

**Step 2: Cognitive interventions**	

Session 5	**Unrealistic expectations on childcare: how the past can influence the present.** *This session reflects on the image of motherhood and the mother. Childcare methods learned from the family of the mother-to-be are compared with those adopted in the partner’s family*.
Session 6	**My inner voice: the missing link.** *The therapist helps participants to understand the impact that thoughts, emotions and behaviors can have on mothers’ everyday life and proposes new ways to deal with negative thoughts, cognitive distortions and/or reasoning errors, by replacing them with more functional thoughts for their well-being*.
Session 7	**Developing a more effective way of thinking.** *In this and in subsequent sessions, the therapist helps participants to focus on techniques to encourage positive thoughts*.
Session 8	**Query/challenge your inner critic.** *Questioning irrational beliefs and negative/critical thoughts*.

**Step 3: Prevention of relapse**	

Session 9	**Putting it all together: “moving forward”.** *Mothers are encouraged to build and use new social networks so that they can refer to them for social support when treatment is concluded*.
Session 10	**Consolidating what I have learned.** *Session to reinforce treatment education and experiences. Mothers can bring their babies*.

**Additional clinical forms with fathers**	

Session 1	**Involving fathers: fathers and post-natal depression.** *In subsequent sessions, fathers are also involved. The therapist provides fathers with information on perinatal depression. This particular session encourages couples to express their feelings about being parents and how they have felt in the past few weeks*.
Session 2	**Couple relationship.** *This session addresses the changes that have occurred in the couple’s relationship since the baby’s birth. Couples are encouraged to communicate and spend quality time together*.
Session 3	**Do it on your own.** *Summarizing the treatment experience and looking at how each couple is dealing with issues related to planning family life with the child*.

**Additional clinical forms with the presence of children: baby H.U.G.S**	

Session 1	**Let’s play! Games and physical contact.** *The therapist introduces neonatal massage technique and talks about the feelings generated by of physical contact. Parents are presented with games for different ages and emphasis is given to the pleasure of proper mother–child interaction and the importance of non-verbal communication*.
Session 2	**Let’s learn something about our child. Observing and understanding his/her signals.** *Activities involving non-verbal interactive behavior are recommended, including stress responses. Parents are encouraged to observe and interpret the child’s non-verbal communication in a playful and fun context. The therapist introduces stimulation activities to identify differences in temperament*.
Session 3	**Let’s analyze our feelings. Parental responses to the signals of their newborn.** *Couples are encouraged to observe the differences between their needs and those of their newborn baby*.

In Italy, following the publication of the Italian language manual (Milgrom, 2003), the actual feasibility and effectiveness of Milgrom’s psychological interventions for the prevention and treatment of postpartum depression has been demonstrated by ISS research (Mirabella et al., 2016).

#### Phase 5: Follow-up

Changes in the patients’ clinical conditions are re-evaluated at three points of follow-up (end of intervention, 6th month and 12th month) using EPDS, PHQ-9, STAI, WHOQOL-BREF, QUITs, MINI-Plus and a clinical interview.

Mothers who are still depressed after the intervention phase are sent to a clinical psychological or psychiatric service.

### Study Outcomes

The data on maternal psychosocial and clinical conditions will be gathered. The primary outcomes are maternal depressive and anxiety symptoms severity. Secondary outcomes are maternal perceived quality of life and infants’ temperament (only for the infants whose mothers participated in the intervention).

### Measures

#### Edinburgh Postnatal Depression Scale

The EPDS (Cox et al., 1987) is the most widely used screening tool for depressive symptoms during the perinatal period (de Moraes et al., 2017; Ukatu et al., 2018). It is a self-rating scale containing 10 items about symptoms of depression such as anhedonia, feelings of guilt, lethargy, sleep disturbance and suicidal ideation. The answer to each item is on a four-point Likert scale with which mothers indicate the frequency with which they have experienced the corresponding symptom during the past 7 days. The total score ranges from 0 to 30, with higher scores indicating more severe depressive symptoms. The internal consistency of the Italian version of the EPDS (Cronbach standardized alpha) was 0.80 (Benvenuti et al., 1999), which is similar to the value reported by Cox in the first validation study (0.87). The sensitivity, specificity, and positive predictive value of the Italian version were recorded as 55.6, 98.9, and 90.9%, respectively, based on a cut-off score ≥12, making it a reliable and valid screening tool for perinatal depression. Furthermore, a recent study (Smith-Nielsen et al., 2018) found that a score of 11 or more is the optimal cut-off for depression according to both DSM-5 (American Psychiatric Association, 2013) and ICD-10 criteria (World Health Organization, 1992).

#### Patient Health Questionnaire-9

The PHQ-9 (Kroenke et al., 2001) is the first choice for depressive symptoms in non-psychiatric primary care settings (Thase, 2016) and the third most common screening tool for postpartum depression (de Moraes et al., 2017). It is a self-rating scale containing nine items about symptoms of depression such as anhedonia and low mood, in conjunction with problems concerning physical activity, appetite, concentration, energy, self-esteem, sleep and suicidal ideation. The answer to each item is on a four-point Likert scale with which mothers indicate the frequency with which they have experienced the corresponding symptom during the past 14 days. The total score ranges from 0 to 27, with higher scores indicating more severe depressive symptoms. During the perinatal period, a score of ≥10 is usually recommended to define the presence of depression (Flynn et al., 2011; Sidebottom et al., 2012; Marcos-Nájera et al., 2018). Furthermore, two of these studies evaluated the factor structure of the PHQ-9, finding that a three-factor (cognitive-affective, somatic, pregnancy-related) model is adequate during pregnancy (Marcos-Nájera et al., 2018). The internal reliability (Cronbach alpha) of the PHQ−9 was 0.86 in an obstetrics−gynecology sample (Kroenke et al., 2001). The sensitivity, specificity, and positive predictive value of the PHQ-9 Italian version (Mazzotti et al., 2003) were recorded as 39, 29, and 93%, respectively, for any depressive syndrome. Furthermore, the PHQ-9 can be used to assess depression severity based on the DSM-5 (American Psychiatric Association, 2013) diagnostic criteria (Spitzer et al., 2014).

#### State-Trait Anxiety Inventory

The STAI (Spielberger et al., 1983) is the most widely used screening tool to identify anxiety in pregnancy (Brunton et al., 2015). It is a self-rating scale containing 40 items divided into two subscales of 20 items each, which evaluate different types of anxiety: the state subscale measures anxiety in the current situation or time period, while the trait subscale measures relatively stable individual aspects of propensity toward elevated anxiety. The answer to each item is on a four-point Likert scale. The total score ranges from 20 to 80, with higher scores indicating more severe anxiety. The STAI internal consistency ranged from 0.86 to 0.95 (Spielberger et al., 1983; Spielberger, 1989) and the Italian version (Pedrabissi and Santinello, 1992) showed psychometric properties consistent with the English version. The adopted cut-off score was ≥ 40, as suggested by the STAI Italian version (Pedrabissi and Santinello, 1992), validated on a non-pregnant population. The construct and content validity of the STAI for pregnant woman has been proven (Gunning et al., 2010). The sensitivity, specificity, and positive predictive value of the STAI for the third trimester of pregnancy were recorded as 80.95, 79.75, and 51.50%, respectively, based on a cut-off score of >40 for both state and trait scales (Grant et al., 2008). A cut-off score of >40 for the postpartum period is recommended (Dennis et al., 2013). The state scale of the STAI is strongly correlated with the EPDS (Meades and Ayers, 2011).

#### Psychosocial and Clinical Assessment Form

The Psychosocial and Clinical Assessment Form (Palumbo et al., 2016) is developed by the Italian National Institute of Health. There are two questionnaires: a pregnancy and a postnatal version. The pregnancy version is composed of 22 items addressing sociodemographic characteristics, information on mental health before pregnancy, stressful life events experienced over the last twelve months, information about the pregnancy and currently perceived family and social support. Whereas the postnatal version has an additional seven items to gather information on childbirth. The resulting psychosocial and clinical risks and protective factors can be weighted and summarized into two different psychological constructs: (a) perceived confidence in personal abilities and own social resources, and (b) perceived psychological vulnerability and ability to cope with the difficulties.

#### Italian Questionnaires of Temperament

The QUIT (Axia, 2002) is a set of questionnaires that are used to measure temperament in infants from the first month up to 11 years of age. This study uses the QUIT 1–12 and 12–36 month Scales. It is a caregiver-report questionnaire validated with reference to Italian culture, containing 55 items investigating six dimensions: attention, inhibition to novelty, motor activities, positive emotionality, negative emotionality and social orientation. The first three dimensions relate to adaptation in general, while the second three relate to specific adaptation to the social world. The answer to each item is on a six-point Likert scale with which mothers indicate their baby’s positive or negative attitude toward the environment, with higher scores indicating greater intensity of a characteristic. Four infant temperamental profiles can be obtained on the basis of the orthogonal dimensions of positive and negative emotionality: emotional temperament, calm temperament, normal temperament, difficult temperament. The QUIT has acceptable internal consistency with Cronbach’s alpha ranging from 0.59 to 0.71 (Axia, 2002), and the Pearson’s correlation coefficient among reports by mothers and fathers ranges from 0.32 to 0.62 (Axia and Weisner, 2002).

#### World Health Organization Quality of Life BREF

The WHOQOL-BRIEF (The Whoqol Group, 1998) is a generic instrument to assess perceived quality of life (QoL). It is a self-rating questionnaire containing 28 items, of which 26 are used to measure four domains related to QoL: physical health, psychological, social relationships and environment. A further two items are used to measure individual perceptions of overall QoL and general health. The answer to each item is on a five-point Likert scale with which mothers indicate their degree of satisfaction/dissatisfaction. Domain scores are calculated by multiplying the mean score of all items within each domain by four and then they are transformed into weighted scores from 0 to 100, with higher scores indicating greater levels of satisfaction. There is no an overall score. The WHOQOL-BRIEF Italian version showed good internal consistency, with Cronbach’s alpha ranging from 0.65 for the social relationships domain to 0.80 for the physical domain (de Girolamo et al., 2000). The WHOQOL-BREF is widely used to measure the QoL of women during the perinatal period (Webster et al., 2010; Nascimento et al., 2011; Mortazavi et al., 2014; Moura et al., 2017).

#### Mini-International Neuropsychiatric Interview-Plus

The MINI-Plus (Sheehan et al., 1998) is a short, structured and standardized diagnostic interview developed to assess the diagnosis of psychotic and mood disorders based on DSM-IV criteria. Its sensitivity, specificity, and reliability are equivalent to those of the Structured Clinical Interview for DSM-IV in different settings (psychiatric and primary care centers) (Sheehan et al., 1998; Amorim, 2000). This study uses the module for depressive disorders, which addresses the frequency, intensity, and duration of depressive symptoms and the degree of the associated distress and impairment. For major depressive disorders, the interrater reliability of the MINI Italian version was 0.96 (Rossi et al., 2004), indicating “excellent agreement.”

### Sample Size

Assuming 10–15% prevalence of perinatal depression as measured by EPDS (Gavin et al., 2005; Melville et al., 2010) the minimum number needed of mothers to be screened is 500.

With regard to the clinical trial on the psychological interventions for perinatal depression and anxiety, the power calculation was based on detecting an effect size of medium magnitude (*d* = 0.55) at the follow-ups on the EPDS among the patients (Cohen, 1988). We calculated that a sample size of 45 patients would result in a power of 0.95 with alpha set at 0.05. To protect against an anticipated loss to follow-up of about 30% (Mirabella et al., 2016), we planned to enroll 65 patients.

### Quality Control

The healthcare centers enter all the data collected with the instruments during the Assessment into an Excel database, prepared by the ISS (baseline + follow-up). Once the centers have entered all the data collected, each researcher (coordinator of the healthcare centers) performs a detailed quality control assessment. After this assessment by each center, the data is sent to the Observatory of Perinatal Clinical Psychology (coordinating center), which repeats the same checks, to ensure that the final database contain no errors and have no missing data.

### Data Analysis Plan

The prevalence of antenatal and postpartum depression and the risk of anxiety will be calculated and compared. Since previous studies have ascertained a higher risk of developing depression in the postpartum period, the antenatal period will be taken as a reference to calculate the odds ratio as a measure of the relative risk of depression between antenatal and postpartum periods, using chi-square as a test for statistical significance.

The sensitivity, specificity and predictive values of the EPDS and the PHQ-9 versus the MINI will be analyzed. Factors associated with ante- and post-partum depression and anxiety risk will be analyzed using a logistic regression model.

With regard to the effectiveness of treatment, standard descriptive statistics will be calculated for all questionnaires. Analysis of variance for repeated measurements will be performed to compare the differences between each initial score obtained with the different administered questionnaires and the scores at T1 (end of treatment), T2 (after 6 months) and T3 (after 12 months) will be evaluated. Bonferroni *post hoc* comparisons will be conducted. Treatment effect will be calculated using Cohen’s d for each of the follow-up times. The Reliable Change Index will be calculated for each time, in order to determine any significant clinical changes occurring in each individual case post treatment.

Data will be analyzed using the Statistical Package for Social Science (SPSS) for Windows version 22.0.

## Ethics and Dissemination

### Description of Risks

There are no risks associated with the participation in any aspect of the described study.

### Informed Consent

Before taking part in the study (which was approved by the Ethical Committee of the Healthcare Centre of Bologna Hospital), all subjects receive information orally and in writing about the content and implications of the study. If they are willing to participate, they have to sign the informed consent form. Recruited subjects are able to withdraw from the study at any time without explanation and with no consequences regarding the care they are receiving.

### Data Protection

All transfers of study data are informed by and undertaken according to the European Parliament and the Council of Europe’s Directive 95/46/EC on the protection of individuals with reference to the processing of personal data. To ensure the security and integrity of data, an appropriate documented standard procedure is established and followed without exception. Any study data that is to be transferred from research sites to the coordinating site are anonymized prior to transfer: each subject is identified with an ID code composed of five alphanumeric characters. All collected data (questionnaires, sheets with test/scale answers, documents, etc.) are marked with the ID code and do not contain any identifying information. Using this ID code, data are entered into the database. Electronic data are stored on a password protected computer and paper material is stored in the locked archive of each healthcare center. Only members of the study team have access to the data. After entering the data into the electronic database and checking data integrity, all paperwork is stored in accordance with the Italian law on privacy (GDPR, May 25, 2018). Personal information about potential and enrolled participants is collected, shared and stored in a manner that protects their confidentiality before, during and after the project.

### Scientific, Clinical, and Social Impact

The project will enable the application of a perinatal depression and anxiety screening procedure that can be developed for the use in different structures, as it requires the collaboration and connection between existing structural and functional resources. The project also recommends the application of early psychological intervention in the health services to prevent perinatal depression and anxiety complications in mothers and also recommends the involvement of fathers and babies. It should be noted that underestimating these disorders could have very negative effects on public health. Early intervention on perinatal depression and anxiety can reduce the direct and indirect costs of damage to the mother in terms of personal, social and working life, and, above all, reduce the direct and indirect costs that may arise due to the impact on child development. For the individual subjects of the study, the expected benefits are the more rapid identification and treatment of anxiety and depressive disorders or other psychiatric disorders, where present, with the consequent possible improvement of mental health outcomes and greater psychological well-being.

Future research should focus on identifying the risk and protective factors with regard to the child’s mental health. Furthermore, due to the principle of transgenerational inheritance (Imbasciati et al., 2011) it is also important to conduct prospective longitudinal studies in order to identify the effects on children who become adults and the consequent possible difficulties they might encounter in their relationship with their own children.

### Strengths and Limitations of This Study

This study presents some strengths and limitations that should be mentioned. The main strengths are: (a) this study provides a new knowledge about the prevalence of perinatal depression and anxiety during both the trimesters of pregnancy and the first four trimesters after birth; (b) the large sample size is based on eleven recruitment centers located throughout Italy, and then it is representative of the pregnant population in Italy; and (c) all mothers were assessed for depression and anxiety through both self-report questionnaires and clinical interviews. The main limitation of the study is that it cannot monitor the timing and patterns of onset, duration, remission and recurrence of anxiety and depression during the entire perinatal period. Another limitation is that this protocol is the absence of a control group or waiting list condition.

## Ethics Statement

The study protocol is approved by the Ethical Committee of the Healthcare Center of Bologna Hospital (Register Number: 0077805, June 27, 2017). The participants provided their written informed consent to participate in this study.

## Author Contributions

This study design is developed in mutual agreement of scientific collaboration between the Observatory of Perinatal Clinical Psychology and the Italian National Institute of Health. LC, GP, and AI contributed equally to the general study design. FM designed the plan of statistical analysis of the study protocol. AG serves primarily as research statistical analysis supervisor. LC and AT from the Observatory coordinate and manage the implementation of the Study in each Healthcare Center. LC, AI, AS, and AT contributed to preparing the manuscript. All authors agreed to submit the final version of the manuscript.

## Conflict of Interest

The authors declare that the research was conducted in the absence of any commercial or financial relationships that could be construed as a potential conflict of interest.

## Footnotes

1 https://www.unibs.it/node/11596

## References

[B1] American Psychiatric Association (2013). *Diagnostic and Statistical Manual of Mental Disorders (DSM-5^®^).* Washington, DC: American Psychiatric Association.

[B2] Amorim P. (2000). Mini international neuropsychiatric interview (MINI): validação de entrevista breve para diagnóstico de transtornos mentais. *Rev. Bras. Psiquiatr.* 22 106–115. 10.1590/S1516-44462000000300003

[B3] Austin M. P. (2004). Antenatal screening and early intervention for “perinatal” distress, depression and anxiety: where to from here? *Arch. Womens. Ment. Health* 7 1–6. 10.1007/s00737-003-0034-4 14963727

[B4] Austin M. P. V. Hadzi-Pavlovic D. Priest S. R. Reilly N. Wilhelm K. Saint K. (2010). Depressive and anxiety disorders in the postpartum period: how prevalent are they and can we improve their detection? *Arch. Womens. Ment. Health* 13 395–401. 10.1007/s00737-010-0153-7 20232218

[B5] Axia G. (2002). *QUIT: Questionari Italiani del Temperamento.* Gardolo: Erikson.

[B6] Axia V. D. Weisner T. S. (2002). Infant stress reactivity and home cultural ecology of Italian infants and families. *Infant Behav. Dev.* 25 255–268. 10.1016/S0163-6383(02)000991

[B7] Banti S. Mauri M. Oppo A. Borri C. Rambelli C. Ramacciotti D. (2011). From the third month of pregnancy to 1 year postpartum. Prevalence, incidence, recurrence, and new onset of depression. Results from the perinatal depression–research & screening unit study. *Compr. Psychiatry* 52 343–351. 10.1016/j.comppsych.2010.08.003 21683171

[B8] Benvenuti P. Ferrara M. Niccolai C. Valoriani V. Cox J. L. (1999). The edinburgh postnatal depression scale: validation for an Italian sample. *J. Affect. Disord.* 53 137–141. 10.1016/S0165-0327(98)00102-5 10360408

[B9] Branchi I. Curley J. P. D’Andrea I. Cirulli F. Champagne F. A. Alleva E. (2013). Early interactions with mother and peers independently build adult social skills and shape BDNF and oxytocin receptor brain levels. *Psychoneuroendocrinology* 38 522–532. 10.1016/j.psyneuen.2012.07.010 22910688 PMC3522751

[B10] Brunton R. J. Dryer R. Saliba A. Kohlhoff J. (2015). Pregnancy anxiety: a systematic review of current scales. *J. Affect. Disord.* 176 24–34. 10.1016/j.jad.2015.01.039 25687280

[B11] Cena (2019). Early screening and prompt intervention to identify and treat maternal mental health problems before and after giving birth. *BMC Res. Prog.* [Preprint]. 10.1186/ISRCTN12733828

[B12] Cirulli F. Berry A. Alleva E. (2003). Early disruption of the mother–infant relationship: effects on brain plasticity and implications for psychopathology. *Neurosci. Biobehav. Rev.* 27 73–82. 10.1016/S0149-7634(03)00010-1 12732224

[B13] Clavenna A. Seletti E. Cartabia M. Didoni A. Fortinguerra F. Sciascia T. (2017). Postnatal depression screening in a paediatric primary care setting in Italy. *BMC Psychiatry* 17:42. 10.1186/s12888-017-1205-6 28122520 PMC5264282

[B14] Cohen J. (1988). *Statistical Power Analysis for the Behavioral Science.* Hillsdale, NJ: Lawrence Erlbaum Associates.

[B15] Cox J. L. Holden J. M. Sagovsky R. (1987). Detection of postnatal depression: development of the 10-item edinburgh postnatal depression scale. *Br. J. Psychiatry* 150 782–786. 10.1192/bjp.150.6.782 3651732

[B16] Currò V. De Rosa E. Maulucci S. Maulucci M. Silvestri M. Zambrano A. (2009). The use of edinburgh postnatal depression scale to identify postnatal depression symptoms at well child visit. *Ital. J. Pediatr.* 35:32. 10.1186/1824-7288-35-32 19863812 PMC2775742

[B17] de Girolamo G. Rucci P. Scocco P. Becchi A. Coppa F. D’Addario A. (2000). Quality of life assessment: validation of the Italian version of the WHOQOL-Brief. *Epidemiol. Psichiatr. Soc.* 9 45–55. 10.1017/S1121189X00007740 10859875

[B18] de Moraes G. P. A. Lorenzo L. Pontes G. A. R. Montenegro M. C. Cantilino A. (2017). Screening and diagnosing postpartum depression: when and how? *Trends Psychiatry Psychother.* 39 54–61. 10.1590/2237-6089-2016-0034 28403324

[B19] Dennis C.-L. Coghlan M. Vigod S. (2013). Can we identify mothers at-risk for postpartum anxiety in the immediate postpartum period using the State-Trait Anxiety Inventory? *J. Affect. Disord.* 150 1217–1220. 10.1016/j.jad.2013.05.049 23764383

[B20] Dennis C.-L. Falah-Hassani K. Shiri R. (2018). Prevalence of antenatal and postnatal anxiety: systematic review and meta-analysis. *Br. J. Psychiatry* 210 315–323. 10.1192/bjp.bp.116.187179 28302701

[B21] Di Venanzio C. Pacitti F. Rossetti M. C. Santarelli V. Gregori E. D’Alfonso A. (2017). Perinatal depression screening and early treatment. *J. Psychopathol.* 23 99–104.

[B22] Dunkel Schetter C. Tanner L. (2012). Anxiety, depression and stress in pregnancy. *Curr. Opin. Psychiatry* 25 141–148. 10.1097/YCO.0b013e3283503680 22262028 PMC4447112

[B23] Earls M. F. Yogman M. W. Mattson G. (2019). AAP committee on psychosocial aspects of child and family health. incorporating recognition and management of perinatal depression into pediatric practice. *Pediatrics* 143:20183259HQ. 10.1542/peds.2018-3259 30559120

[B24] Elisei S. Lucarini E. Murgia N. Ferranti L. Attademo L. (2013). Perinatal depression: a study of prevalence and of risk and protective factors. *Psychiatr. Danub.* 25(Suppl. 2) S258–S262. 23995189

[B25] Erickson N. L. Gartstein M. A. Dotson J. A. W. (2017). Review of prenatal maternal mental health and the development of infant temperament. *J. Obst. Gynecol. Neonatal Nurs.* 46 588–600. 10.1016/j.jogn.2017.03.008 28571833

[B26] Falah-Hassani K. Shiri R. Dennis C.-L. (2016). Prevalence and risk factors for comorbid postpartum depressive symptomatology and anxiety. *J. Affect. Disord.* 198 142–147. 10.1016/j.jad.2016.03.010 27016657

[B27] Field T. (2018). Postnatal anxiety prevalence, predictors and effects on development: a narrative review. *Infant Behav. Dev.* 51 24–32. 10.1016/j.infbeh.2018.02.005 29544195

[B28] Flynn H. A. Sexton M. Ratliff S. Porter K. Zivin K. (2011). Comparative performance of the edinburgh postnatal depression scale and the patient health questionnaire-9 in pregnant and postpartum women seeking psychiatric services. *Psychiatry Res.* 187 130–134. 10.1016/j.psychres.2010.10.022 21122923

[B29] Galbally M. Lewis A. J. (2017). Depression and parenting: the need for improved intervention models. *Curr. Opin. Psychol.* 15 61–65. 10.1016/j.copsyc.2017.02.008 28813270

[B30] Gavin N. I. Gaynes B. N. Lohr K. N. Meltzer-Brody S. Gartlehner G. Swinson T. (2005). Perinatal depression. *Obstet. Gynecol.* 106 1071–1083. 10.1097/01.AOG.0000183597.31630.db 16260528

[B31] Ghaedrahmati M. Kazemi A. Kheirabadi G. Ebrahimi A. Bahrami M. (2017). Postpartum depression risk factors: a narrative review. *J. Educ. Health Promot.* 6:60. 10.4103/jehp.jehp_9_16 28852652 PMC5561681

[B32] Giardinelli L. Innocenti A. Benni L. Stefanini M. C. Lino G. Lunardi C. (2012). Depression and anxiety in perinatal period: prevalence and risk factors in an Italian sample. *Arch. Womens Ment. Health* 15 21–30. 10.1007/s00737-011-0249-8 22205237

[B33] Girardi P. Pompili M. Innamorati M. Serafini G. Berrettoni C. Angeletti G. (2011). Temperament, post-partum depression, hopelessness, and suicide risk among women soon after delivering. *Women Health* 51 511–524. 10.1080/03630242.2011.583980 21797682

[B34] Glover V. (2015). Prenatal stress and its effects on the fetus and suicide risk among women soon after delivering. *Perinat. Program. Neurodev.* 10 269–283. 10.1007/978-1-4939-1372-5_13 25287545

[B35] Grant K.-A. McMahon C. Austin M.-P. (2008). Maternal anxiety during the transition to parenthood: a prospective study. *J. Affect. Disord.* 108 101–111. 10.1016/j.jad.2007.10.002 18001841

[B36] Gunning M. D. Denison F. C. Stockley C. J. Ho S. P. Sandhu H. K. Reynolds R. M. (2010). Assessing maternal anxiety in pregnancy with the State-Trait Anxiety Inventory (STAI): issues of validity, location and participation. *J. Reprod. Infant Psychol.* 28 266–273. 10.1080/02646830903487300

[B37] Hahn-Holbrook J. Cornwell-Hinrichs T. Anaya I. (2018). Economic and health predictors of national postpartum depression prevalence: a systematic review, meta-analysis, and meta-regression of 291 studies from 56 countries. *Front. Psychiatry* 8:248. 10.3389/fpsyt.2017.00248 29449816 PMC5799244

[B38] Imbasciati A. (2006). *Constructing a Mind: A New Base for Psychoanalytic Theory.* London: Routledge.

[B39] Imbasciati A. Cena L. (2015a). *Psicologia Clinica Perinatale per le Professioni Sanitarie E Psicosociali. Vol. I. Neonato e Radici Della Salute Mentale.* Milano: Franco Angeli.

[B40] Imbasciati A. Cena L. (2015b). *Psicologia Clinica Perinatale per le Professioni Sanitarie e Psicosociali. Vol. 2: Genitorialità e Origini Della Mente del Bambino.* Milano: FrancoAngeli.

[B41] Imbasciati A. Cena L. (2017). *Psicologia Clinica Perinatale – Neuroscienze e Psicoanalisi.* Milano: Franco Angeli.

[B42] Imbasciati A. Cena L. (2018). *Il futuro dei Primi Mille Giorni di Vita.* Milano: Franco Angeli.

[B43] Imbasciati A. Dabrassi F. Cena L. (2007). *Psicologia Clinica Perinatale.* Padova: Piccin.

[B44] Imbasciati A. Dabrassi F. Cena L. (2011). *Psicologia Clinica Perinatale per il Futuro Individuo: Un Uomo Transgenerazionale.* Torino: Ed Express.

[B45] Kendig S. Keats J. P. Hoffman M. C. Kay L. B. Miller E. S. Moore Simas T. A. (2017). Consensus bundle on maternal mental health: perinatal depression and anxiety. *J. Obstet. Gynecol. Neonatal Nurs.* 46 272–281. 10.1016/j.jogn.2017.01.001 28190757

[B46] Kim-Cohen J. Moffitt T. E. Taylor A. Pawlby S. J. Caspi A. (2005). Maternal depression and children’s antisocial behavior. *Arch. Gen. Psychiatry* 62 173–181. 10.1001/archpsyc.62.2.173 15699294

[B47] Korja R. Nolvi S. Kataja E.-L. Scheinin N. Junttila N. Lahtinen H. (2018). The courses of maternal and paternal depressive and anxiety symptoms during the prenatal period in the FinnBrain Birth Cohort study. *PLoS One* 13:e0207856. 10.1371/journal.pone.0207856 30557345 PMC6296666

[B48] Kroenke K. Spitzer R. L. Williams J. B. W. (2001). The PHQ-9: validity of a brief depression severity measure. *J. Gen. Intern. Med.* 16 606–613. 10.1046/j.1525-1497.2001.016009606.x 11556941 PMC1495268

[B49] Leach L. Poyser C. Fairweather-Schmidt K. (2017). Maternal perinatal anxiety: a review of prevalence and correlates. *Clin. Psychol.* 21 4–19. 10.1111/cp.12058

[B50] Lecrubier Y. Sheehan D. Weiller E. Amorim P. Bonora I. Harnett Sheehan K. (1997). The mini international neuropsychiatric interview (MINI). a short diagnostic structured interview: reliability and validity according to the CIDI. *Eur. Psychiatry* 12 224–231. 10.1016/S0924-9338(97)83296-8

[B51] Marcos-Nájera R. Le H.-N. Rodríguez-Muñoz M. F. Olivares Crespo M. E. Izquierdo Mendez N. (2018). The structure of the patient health questionnaire-9 in pregnant women in Spain. *Midwifery* 62 36–41. 10.1016/j.midw.2018.03.011 29653416

[B52] Mauri M. Oppo A. Montagnani M. S. Borri C. Banti S. Camilleri V. (2010). Beyond “postpartum depressions”: specific anxiety diagnoses during pregnancy predict different outcomes. *J. Affect. Disord.* 127 177–184. 10.1016/j.jad.2010.05.015 20554326

[B53] Mazzotti E. Fassone G. Picardi A. Sagoni E. Ramieri L. Lega I. (2003). The patient health questionnaire (PHQ) for the screening of psychiatric disorders: a validation study versus the structured clinical interview for DSM-IV axis I (SCID-I). *Ital. J. Psychopathol.* 9 235–242.

[B54] Meades R. Ayers S. (2011). Anxiety measures validated in perinatal populations: a systematic review. *J. Affect. Disord.* 133 1–15. 10.1016/j.jad.2010.10.009 21078523

[B55] Melville J. L. Gavin A. Guo Y. Fan M.-Y. Katon W. J. (2010). Depressive disorders during pregnancy. *Obstet. Gynecol.* 116 1064–1070. 10.1097/AOG.0b013e3181f60b0a 20966690 PMC3068619

[B56] Meneghetti A. (2007). *System and Personality.* Rome: Ontopsicologia Editrice.

[B57] Meneghetti A. (2011). *Project human being.* Rome: Ontopsicologia Editrice.

[B58] Milgrom J. (2003). *Depressione Postnatale. Ricerca, Prevenzione e Strategie di Intervento Psicologico.* Trento: Erikson.

[B59] Milgrom J. Ericksen J. McCarthy R. Gemmill A. W. (2006). Stressful impact of depression on early mother–infant relations. *Stress Health* 22 229–238. 10.1002/smi.1101

[B60] Milgrom J. Gemmil A. W. (2015). *Identifying Perinatal Depression and Anxiety: Evidence-Based Practice in Screening, Psychosocial Assessment and Management.* Malden, MA: John Wiley & Sons. 10.1080/02646838.2017.1315633 29517306

[B61] Milgrom J. Gemmill A. W. Ericksen J. Burrows G. Buist A. Reece J. (2015a). Treatment of postnatal depression with cognitive behavioural therapy, sertraline and combination therapy: a randomised controlled trial. *Aust. New Zeal. J. Psychiatry* 49 236–245. 10.1177/0004867414565474 25586754

[B62] Milgrom J. Holt C. Holt C. J. Ross J. Ericksen J. Gemmill A. W. (2015b). Feasibility study and pilot randomised trial of an antenatal depression treatment with infant follow-up. *Arch. Womens. Ment. Health* 18 717–730. 10.1007/s00737-015-0512-5 25709044

[B63] Milgrom J. Martin P. R. Negri L. M. (1999). *Treating Postnatal Depression: A Psychological Approach for Health Care Practitioners.* Chichester: Wiley.

[B64] Milgrom J. Schembri C. Ericksen J. Ross J. Gemmill A. W. (2011). Towards parenthood: an antenatal intervention to reduce depression, anxiety and parenting difficulties. *J. Affect. Disord.* 130 385–394. 10.1016/j.jad.2010.10.045 21112641

[B65] Mirabella F. Michielin P. Piacentini D. Veltro F. Barbano G. Cattaneo M. (2016). Efficacia di un intervento psicologico rivolto a donne positive allo screening per depressione post partum. *Riv. Psichiatr.* 51 260–269. 10.1708/2596.26728 27996986

[B66] Misri S. Swift E. (2015). Generalized anxiety disorder and major depressive disorder in pregnant and postpartum women: maternal quality of life and treatment outcomes. *J. Obstet. Gynaecol. Canada* 37 798–803. 10.1016/S1701-2163(15)30150-X 26605449

[B67] Mortazavi F. Mousavi S. A. Chaman R. Khosravi A. (2014). Maternal quality of life during the transition to motherhood. *Iran. Red Crescent Med. J.* 16:e8443. 10.5812/ircmj.8443 25031866 PMC4082526

[B68] Moura M. R. S. Araújo C. G. A. Prado M. M. Paro H. B. M. S. Pinto R. M. C. Abdallah V. O. S. (2017). Factors associated with the quality of life of mothers of preterm infants with very low birth weight: a 3-year follow-up study. *Qual. Life Res.* 26 1349–1360. 10.1007/s11136-016-1456-6 27888392

[B69] Nascimento S. Surita F. Parpinelli M. Siani S. Pinto E. Silva J. (2011). The effect of an antenatal physical exercise programme on maternal/perinatal outcomes and quality of life in overweight and obese pregnant women: a randomised clinical trial. *BJOG Int. J. Obstet. Gynaecol.* 118 1455–1463. 10.1111/j.1471-0528.2011.03084.x 21895947

[B70] O’Connor E. Rossom R. C. Henninger M. Groom H. C. Burda B. U. (2016). Primary care screening for and treatment of depression in pregnant and postpartum women. *JAMA* 315 388–406. 10.1001/jama.2015.18948 26813212

[B71] Okagbue H. I. Adamu P. I. Bishop S. A. Oguntunde P. E. Opanuga A. A. Akhmetshin E. M. (2017). Systematic review of prevalence of antepartum depression during the trimesters of pregnancy. *Open Access Maced. J. Med. Sci.* 7 1555–1560. 10.3889/oamjms.2019.270 31198472 PMC6542400

[B72] Palumbo G. Mirabella F. Barbano G. Cattaneo M. Gigantesco A. (2018). “Dalla ricerca alla pratica: prevenzione e intervento precoce per il rischio di depressione post partum,” in *Il Futuro dei Primi Mille Giorni di Vita*, Imbasciati A. Cena L. (Milan: FrancoAngeli).

[B73] Palumbo G. Mirabella F. Cascavilla I. Del Re D. Romano G. Gigantesco A. (2016). *Prevenzione e Intervento Precoce per il Rischio di Depressione Post Partum. (Rapporti ISTISAN 16/31).* Rome: Istituto Superiore di Sanità.

[B74] Palumbo G. Mirabella F. Imbasciati A. Cena L. Barbano G. Cattaneo M. (2017). Prevenzione della sofferenza psichica perinatale. *Not. dell’Istituto Super. Sanità* 30 3–7.

[B75] Parfitt Y. Ayers S. (2014). Transition to parenthood and mental health in first-time parents. *Infant Ment. Health J.* 35 263–273. 10.1002/imhj.21443 25798480

[B76] Pedrabissi L. Santinello M. (1992). *STAI: State-Trait Anxiety Inventory—Forma Y—Manual.* Firenze: Organizzazioni Speciali.

[B77] Reck C. Struben K. Backenstrass M. Stefenelli U. Reinig K. Fuchs T. (2008). Prevalence, onset and comorbidity of postpartum anxiety and depressive disorders. *Acta Psychiatr. Scand.* 118 459–468. 10.1111/j.1600-0447.2008.01264.x 18840256

[B78] Riva Crugnola C. Ierardi E. Ferro V. Gallucci M. Parodi C. Astengo M. (2016). Mother-infant emotion regulation at three months: the role of maternal anxiety, depression and parenting stress. *Psychopathology* 49 285–294. 10.1159/000446811 27410251

[B79] Robinson G. E. Stewart D. E. (2001). “Postpartum disorders,” in *Psychological Aspects of Women’s Health Care*, 2nd Edn, eds Stotland N. L. Stewart D. E. (Washington. DC: American Psychiatric Press), 117–139.

[B80] Rossi A. Alberio R. Porta A. Sandri M. Tansella M. Amaddeo F. (2004). The reliability of the mini-international neuropsychiatric interview – Italian version. *J. Clin. Psychopharmacol.* 24 561–563. 10.1097/01.jcp.0000139758.03834.ad 15349020

[B81] Santos I. S. Tavares B. F. Munhoz T. N. Manzolli P. de Ávila G. B. Jannke E. (2016). Patient health questionnaire-9 versus Edinburgh postnatal depression scale in screening for major depressive episodes: a cross-sectional population-based study. *BMC Res. Notes* 9:453. 10.1186/s13104-016-2364-0 27677844 PMC5037593

[B82] Sheehan D. V. Lecrubier Y. Sheehan K. H. Amorim P. Janavs J. Weiller E. (1998). The mini-international neuropsychiatric interview (M.I.N.I.): the development and validation of a structured diagnostic psychiatric interview for DSM-IV and ICD-10. *J. Clin. Psychiatry* 59 22–33. 9881538

[B83] Shonkoff M. Duncan G. J. Yoshikawa H. (2019). *Maternal Depression can Undermine the Development of Young Children. Centre on the Developing Child.* Cambridge, MA: Harvard University.

[B84] Sidebottom A. C. Harrison P. A. Godecker A. Kim H. (2012). Validation of the patient health questionnaire (PHQ)-9 for prenatal depression screening. *Arch. Womens Ment. Health* 15 367–374. 10.1007/s00737-012-0295-x 22983357

[B85] Sipsma H. L. Callands T. Desrosiers A. Magriples U. Jones K. Albritton T. (2016). Exploring trajectories and predictors of depressive symptoms among young couples during their transition to parenthood. *Matern. Child Health J.* 20 2372–2381. 10.1007/s10995-016-2064-3 27541145 PMC8728795

[B86] Smith-Nielsen J. Matthey S. Lange T. Væver M. S. (2018). Validation of the edinburgh postnatal depression scale against both DSM-5 and ICD-10 diagnostic criteria for depression. *BMC Psychiatry* 18:393. 10.1186/s12888-018-1965-7 30572867 PMC6302501

[B87] Spielberger C. D. (1989). *State-Trait Anxiety Inventory: Bibliography*, 2nd Edn Palo Alto, CA: Consulting Psychologists Press.

[B88] Spielberger C. D. Gorsuch R. L. Luschene R. Vagg P. R. Jacobs G. A. (1983). *Manual for the State-Trait Anxiety Inventory STAI (Form Y)(“Self-Evaluation Questionnaire”).* Palo Alto, CA: Consulting Psychologists Press.

[B89] Spitzer R. L. Williams J. B. W. Kroeneke K. (2014). Test review: patient health questionnaire–9 (PHQ-9). *Rehabil. Couns. Bull.* 57 246–248. 10.1177/0034355213515305

[B90] Stefana A. Lavelli M. (2017). Parental engagement and early interactions with preterm infants during the stay in the neonatal intensive care unit: protocol of a mixed-method and longitudinal study. *BMJ Open* 7:e013824. 10.1136/bmjopen-2016-013824 28153932 PMC5293994

[B91] Suri R. Lin A. S. Cohen L. S. Altshuler L. L. (2014). Acute and long-term behavioral outcome of infants and children exposed in utero to either maternal depression or antidepressants. *J. Clin. Psychiatry* 75 e1142–e1152. 10.4088/JCP.13r08926 25373125

[B92] Thase M. E. (2016). Recommendations for screening for depression in adults. *JAMA* 315 349–350. 10.1001/jama.2015.18406 26813206

[B93] The Whoqol Group. (1998). Development of the world health organization WHOQOL-BRIEF quality of life assessment. *Psychol. Med.* 28 551–558. 10.1017/s0033291798006667 9626712

[B94] Ukatu N. Clare C. A. Brulja M. (2018). Postpartum depression screening tools: a review. *Psychosomatics* 59 211–219. 10.1016/j.psym.2017.11.005 29396166

[B95] Underwood L. Waldie K. D’Souza S. Peterson E. R. Morton S. (2016). A review of longitudinal studies on antenatal and postnatal depression. *Arch. Womens Ment. Health* 19 711–720. 10.1007/s00737-016-0629-1 27085795

[B96] van der Waerden J. Galéra C. Larroque B. Saurel-Cubizolles M.-J. Sutter-Dallay A.-L. Melchior M. (2015). Maternal depression trajectories and children’s behavior at age 5 years. *J. Pediatr.* 166 1440.e–1448.e. 10.1016/j.jpeds.2015.03.002 25866387

[B97] Vizzini L. Popovic M. Zugna D. Vitiello B. Trevisan M. Pizzi C. (2018). Maternal anxiety, depression and sleep disorders before and during pregnancy, and preschool ADHD symptoms in the NINFEA birth cohort study. *Epidemiol. Psychiatr. Sci.* 28 521–531. 10.1017/S2045796018000185 29665879 PMC6998915

[B98] Webster J. Nicholas C. Velacott C. Cridland N. Fawcett L. (2010). Validation of the WHOQOL-BREF among women following childbirth. *Aust. New Zeal. J. Obstet. Gynaecol.* 50 132–137. 10.1111/j.1479-828X.2009.01131.x 20522068

[B99] World Health Organization (1992). *The ICD-10 Classification of Mental and Behavioural Disorders: Clinical Descriptions and Diagnostic Guidelines.* Geneva: World Health Organization.

[B100] Yawn B. P. Pace W. Wollan P. C. Bertram S. Kurland M. Graham D. (2009). Concordance of edinburgh postnatal depression scale (EPDS) and patient health questionnaire (PHQ-9) to assess increased risk of depression among postpartum women. *J. Am. Board Fam. Med.* 22 483–491. 10.3122/jabfm.2009.05.080155 19734393

[B101] Yeaton-Massey A. Herrero T. (2019). Recognizing maternal mental health disorders. *Curr. Opin. Obstet. Gynecol.* 31 116–119. 10.1097/GCO.0000000000000524 30694850

[B102] Zhong Q. Gelaye B. Rondon M. Sánchez S. E. García P. J. Sánchez E. (2014). Comparative performance of patient health questionnaire-9 and edinburgh postnatal depression scale for screening antepartum depression. *J. Affect. Disord.* 162 1–7. 10.1016/j.jad.2014.03.028 24766996 PMC4040145

